# Working conditions and attitudes towards work: the case of Portuguese youth from Braga

**DOI:** 10.3389/fsoc.2024.1356527

**Published:** 2024-02-14

**Authors:** Eduardo Duque, José F. Durán Vázquez

**Affiliations:** ^1^Faculty of Philosophy and Social Sciences, Universidade Católica Portuguesa & Communication and Society Research Centre (CECS) of the University of Minho, Braga, Portugal; ^2^Department of Sociology, Political Science and Management and Philosophy, Universidade of Vigo, Vigo, Spain

**Keywords:** youth, work, attitudes, identity, Portugal

## Abstract

The purpose of this study is to explore the working conditions and attitudes towards work among the youth of Braga, Portugal. Focusing on residents of the Municipality of Braga born between 1990 and 2000, we employed a quantitative approach using a non-probabilistic convenience sampling method, with a sample size of 406 people. The survey, conducted through questionnaires, reveals a complex interplay between the youth’s work attitudes and their conditions. Despite the predominance of precarious employment, their work experiences and expectations do not entirely reflect this instability. Our findings suggest a shift in the role of work in the youths’ identity compared to previous generations. This shift, common in post-industrial societies, shows that their identity is increasingly tied to the sociability their work enables and other life spheres where they seek fulfilment, as evidenced by how they utilize their earnings from these occupations.

## 1 Introduction

The world of work has experienced profound changes in recent decades, which have resulted in increasingly uncertain and unstable work trajectories (Piore and Sabel, 1990; Gorz, 1997; Bauman, 2000; Beck, 2006). This fact has particularly affected the younger population, with increasingly precarious employment relationships (Pais, 2012; Benedicto, 2016; Artegui, 2017; Nico, 2017; Nada et al., 2022).

However, these working conditions, and particularly those of the younger population, the subject of this work, have not been accompanied by a perception of work that reproduces this precariousness, because for a part of this youth, and particularly those who have disadvantages jobs, the world of work no longer constitutes a central aspect of their identity, as was the case with young people of previous generations (Brooks, 2001; Florida, 2004; Callejo, 2009; Lloyd, 2012; Durán Vázquez and Duque, 2018; Farrugia, 2018; Balderson et al., 2020).

Analysing the working world of youth implies, therefore, relating objective working conditions with subjective attitudes towards work; ways of working and work culture. A relationship that becomes complex, because not always, as has been said, working conditions, which in objective terms can be defined as precarious, appear linked to negative attitudes that reflect said precariousness. Because work can be understood as an instrumental reality that provides economic income and a certain stability, which enables the integration of subjects in other spheres of life, which they, perhaps, consider more relevant for the construction of their identities (Ramos Torre, 2008; Garner et al., 2009; Balderson et al., 2020). On other occasions, on the contrary, work can be considered a much more rewarding experience, when the contractual conditions are much better and the work activity is linked to personal tastes, thus enabling the personal fulfilment of those who perform it. This expressive dimension of work blurs the distinction between work and life, because life is work and work is life (Brooks, 2001; Florida, 2004; Valenzuela et al., 2015).

In both scenarios, work cannot be comprehended solely in terms of performing a specific task, whose more or less monotonous and routine or innovative and creative nature, would necessarily produce a world of work lacking meaning, or, on the contrary, filled with it. As Max Weber has shown, the most monotonous and routine dedication to the task to which one dedicates himself daily can harbour the greatest meaning, when the work is linked to a greater purpose that exceeds the meaning of said task (Weber, 1998, p. 172–173). What’s more, sometimes even the task can be very painful for those who perform it, and yet their work world appears loaded with meaning, if it is in line with certain values linked to their respective life worlds (Willis, 1988; Sennett, 2001).

In the present work, which aims to describe and analyse the working conditions, perceptions and attitudes towards work of Portuguese youth in Braga’s city area, in Portugal, it will be shown, precisely, that some mostly precarious working conditions, like those that Portuguese youth have usually had (Jalali et al., 2020; Tavares et al., 2021; Nada et al., 2022), do not necessarily imply that their perceptions and attitudes towards work are the manifestation of said precariousness.

Next, we will describe the methodology and techniques that we have used to carry out this research, and then show the results we have reached.

## 2 Materials and methods

The methodology that we have used to develop this research has been quantitative, based on the questionnaire survey technique, in which various questions related to the socio-professional situation and work attitudes of the surveyed population were asked.

To prepare the questionnaire we have established a series of dimensions, linked to the objectives set in the research, related to a series of indicators. These dimensions have allowed us to describe and analyse a series of aspects, namely, the relationships that individuals maintain with their respective jobs, perception and satisfaction at work, the influence of work on the construction of identity, the relationship between the educational and working world, and between work and personal life, particularly that related to the field of consumption. All these aspects were crossed with the sociodemographic characteristics of the population, to achieve a more integrated approach, thus being able to observe how the different sociocultural and institutional factors influence the work experience of the youth that is the subject of our research.

The questionnaire survey was carried out in the period between September and December 2021. The population under study includes residents of the municipality of Braga, of both genders, between 20 and 30 years old.

To carry out this study, we have chosen a data collection system through personal and online contact. First, we conducted personal interviews with young residents in the municipality of Braga. We also requested the Municipal Chamber of Braga and different “*Civil Parish Boards*” of the aforementioned municipality to distribute the questionnaire to the young population under study. Likewise, we asked different young people who were interested in participating in the research to distribute the questionnaire among their networks of acquaintances via e-mail. This method resulted in the creation of a non-probabilistic convenience sample.

The choice of this non-probabilistic convenience sample was based on its effectiveness and efficiency in quickly reaching a specific group. Unlike probability samples, where each member of the population has a known probability of being selected, convenience sampling depends on the availability and interest of participants (Duque and Calheiros, 2017). Although this approach does not allow for traditional statistical inference due to the lack of random representation, it can provide valuable insights. This is because the sample consists of a segment of the public that, to a certain extent, can reflect relevant aspects of society. Thus, within the specific context and limitations of this technique, it is possible to draw significant conclusions that can inform the trends, perceptions or behaviours of a given group.

Once the data from the in-person survey and the online survey were analysed, it was observed that there were no significant differences in the use of these two types of data. This finding is consistent with what has been observed by other authors (Hewson and Charlton, 2005; Vallejo et al., 2007; Spek et al., 2008; Campos et al., 2011), who also agree that the responses received online are equivalent to those obtained in person. Hence, we decided to treat both data together.

In this way, we received 520 questionnaires, of which 406 corresponded to the criteria established to identify the population under study, those residing in the Municipality of Braga born between 1990 and 2000.

The demographic characteristics of the sample population, segmented according to sex, age, level of education and parental education, are as follows. The age of the people who make up the sample is between 21 and 30 years old. Regarding their academic training, 49% of the surveyed population completed secondary education, 28% have a university degree, 16% a master’s degree, 6% a technological specialization degree, and 1% a Ph.D. Regarding the academic training of the parents, 67% have completed basic education, 13% have completed secondary education, 11% have a university degree, 5% have a master’s degree and 1% have a doctorate. Parents have, therefore, a lower educational level than their children.

## 3 Results

### 3.1 The youth of Braga in the context of Portuguese youth

For several decades now, Portuguese youth have had a difficult employment situation, dominated by precariousness and instability (Nelson et al., 1989; Cabral and Pais, 1998; Smithson et al., 1998; Nico, 2017; Sagnier and Morell, 2021; Nada et al., 2022). This situation of job insecurity has been accompanied, however, by a gradual and substantial increase in the academic training of young people (Sagnier and Morell, 2021; Nada et al., 2022).

The youth of Braga between 20 and 29 years old present characteristics not very different from Portuguese youth. Specifically, the municipality of Braga has a significantly higher percentage of young people of this age than the rest of Portugal (11.8% compared to 10.7% according to the 2021 Census). If we differentiate within the youth of Braga those who are between 20 and 24 years old from those who are between 25 and 29 years old, we see that the first group is significantly more numerous than the second (50.4% compared to 49.6%).

Regarding their level of schooling, 5 out of every 10 young people between 20 and 29 years old have completed compulsory schooling (46.6%), 4 out of every 10 have a university degree (40%), 10.3% have basic education, and only 0.7% state that they have no studies. These figures, especially those referring to higher education, are significantly higher than those of Portugal as a whole.

### 3.2 The employment situation of the youth of Braga

According to the 2021 Census, 6 out of every 10 young residents in Braga are working (59.2%), 2 out of every 10 are studying (23.4%), while 1 out of every 10 is unemployed (7.9%). The majority of the income available to these young people comes precisely from work (58.1%), income that is substantially compensated by those that come from the family (30.8%).

The majority of young people who work do so as salaried employees, specifically 9 out of 10 (85.5%), while only 5.6% of these young people work on their own account.

If we compare work with the level of education, we can say that of the 60% of young people in Braga who say they have a job, 41.2% have completed secondary education and 43.7% have completed university education.

The majority of Braga youth between 20 and 29 years old, who are unemployed, say they are looking for a job (82.4%); many fewer unemployed young people state that they have never been employed and are therefore looking for their first job (17.6%). These percentages are not very different from those of Portugal as a whole. If we link unemployed youth with their level of education, we see that 49% have secondary education and 32.4% have higher education.

If we analyse the employment situation of the youth of Braga, we can see that of the young people between 21 and 31 years old, 53.7% work full-time, and 12.8% work part-time. Regarding other young people, 9.4% say that they are not currently working, but have previously worked full-time, while 11.9% say that, although they are not working now, they have previously worked part-time. On the contrary, 12.3% of young people say they have never worked.

From a gender point of view, work experience is significantly higher in men than in women, since while 93.8% of men report that they are currently employed or that, although they are not working now, they have had a job in the past, this figure drops to 82.1% in the case of women.

As might be expected, it is the older young people (26–31 years old) who have more work experience, since 77.6% of them say they are working, of which the majority (66.9%) do it full time, compared to 55.2% of those between 21 and 25 years old, of whom 43.3% say they work full time. In this last age group, there are also the highest figures of those who say they have never worked before (22.9%), compared to 2% of those between 21 and 25 years old.

If we relate the employment situation to the level of schooling, it is observed that young people who have a university degree generally have a higher level of labour insertion than those who have a degree in secondary or technical education (72.8% compared to 61.3%).

Young people with secondary and technical studies are the ones with the highest percentage of having a full-time job, surpassing in this aspect those with a university degree (55.9 and 51.1%, respectively).

**Figure 1:**
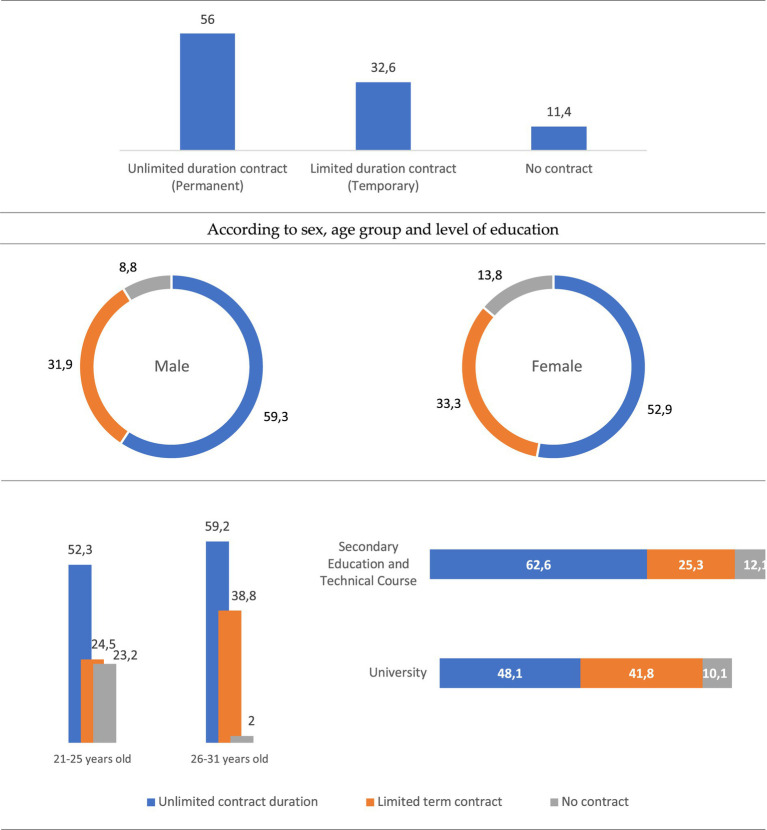
Type of employment contract (%). Source: own preparation based on a survey of young people in the Municipality of Braga.

From the point of view of job stability, 6 out of 10 young people say they have a permanent contract, 3 out of 10 say they have a temporary employment contract, while 1 in 10 state that they do not have an employment contract, without significant variations being seen between genders. However, it is young people between 21 and 25 years old who have a more unstable employment situation, since 23.2% of them say that they do not have any employment contract, a percentage that in the case of young people between 26 and 31 years old is much lower, reducing to 2%, with many more young people in this age group saying they have an indefinite temporary contract, specifically 4 out of every 10. All of this highlights the age at which Braga’s youth are fully incorporated into the labour market (Figure 1). However, as these figures also show, this incorporation is still largely precarious.

Young people who have a degree in secondary or technical education are those who have a higher percentage of indefinite-term contracts (62.6%) compared to those who have a university degree (48.1%) (Figure 1). All of this shows that having a university degree does not translate into greater professional stability, at least compared to secondary and technical education.

**Figure 2:**
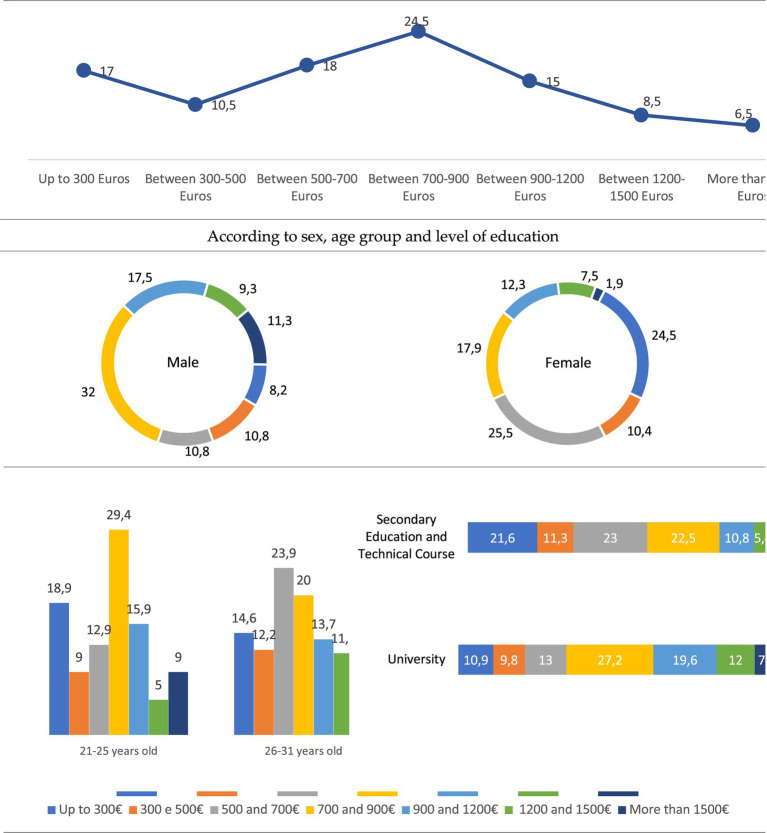
Young people’s income (%). Source: own preparation based on a survey of young people in the Municipality of Braga.

Another indicator of the greater or lesser precariousness of the employment situation of the youth of Braga is the salary level. Well, 7 out of 10 young people say they have a salary of less than 900 euros per month, 3 out of 10 say they earn more than 900 euros per month, 15% of the young people surveyed say they receive between 900 and 1,200 euros, 8.5% between 1,200 and 1,500, and 6.5% say they have a salary of more than 1,500 euros per month. Young people with university studies are the ones who in the highest percentage say they have a salary greater than 900 euros per month (39.2% compared to 21.6% of those with secondary and technical studies) (Figure 2).

**Figure 3:**
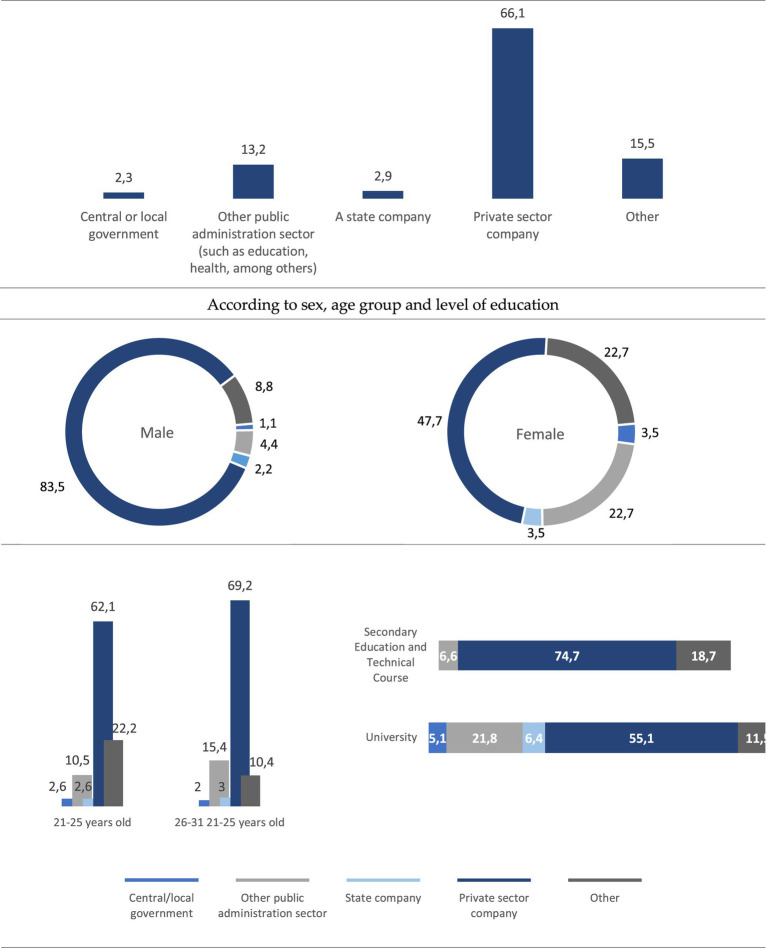
The organization\company for whom he/she works or worked (%). Source: own preparation based on a survey of young people in the Municipality of Braga.

**Figure 4:**
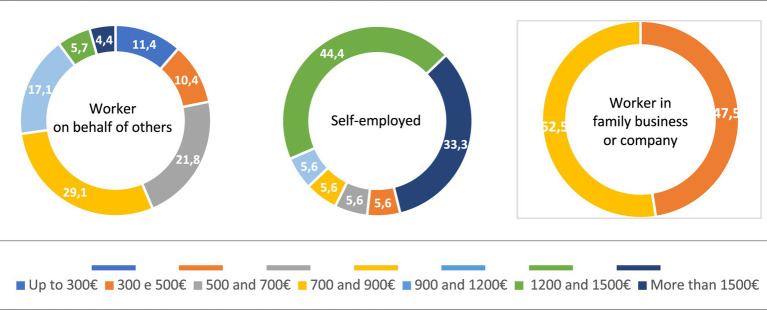
Income of young people, according to type of work (%). Source: own preparation based on a survey of young people in the Municipality of Braga.

The majority of the people we surveyed work, or have worked, in the private sector (66.1%), while 18.4% are or were employed in the public sector. If we analyse this issue according to the level of education, we see that it is young people with secondary and technical studies who in the highest percentage are employed in the private sector (74.7%), while this percentage is reduced to 55.1% in those who have higher education (Figure 3). If we relate these data to the salary level, we observe that the majority of young employees (72.7%) claim to have a salary of less than 900 euros per month. Very different percentage from that of young people who work on their own, since 74.7% say they earn more than 900 euros per month, 44.4% between 1,200 and 1,500 euros and 33.3% say they earn more than 1,500 euros per month (Figure 4). For their part, young people who work in the public sector receive relatively higher salaries than those who work in the private sector, since 6 out of 10 claim to earn between 900 and 1,200 euros.

In summary, we can say that the salary level of the majority of Braga’s youth is low, with monthly salaries of less than 900 euros. All of this is even more significant if we consider that 58.7% of young people who work full-time claim to earn less than 900 euros per month. A situation that is even more aggravated if one considers that while the educational qualifications of Braga’s youth have only increased in recent decades, their salary level remains relatively low both in Braga and in Portugal as a whole (Sagnier and Morell, 2021).

Up to this point we have described the employment situation of the youth of Braga in relation to their salary level, their types of contracts and the sectors in which they are employed. However, as has already been pointed out, this employment situation does not in itself explain the perception that young people have of work. This perception is also influenced by their work culture. To understand how the work situation is related to the work culture, it is necessary to first describe the attitudes towards work of the youth of Braga. This will enable us to discuss how work culture interacts with the work situation of the youth who are the focus of our study.

### 3.3 Perception of work and work attitudes of the youth of Braga

**Figure 5:**
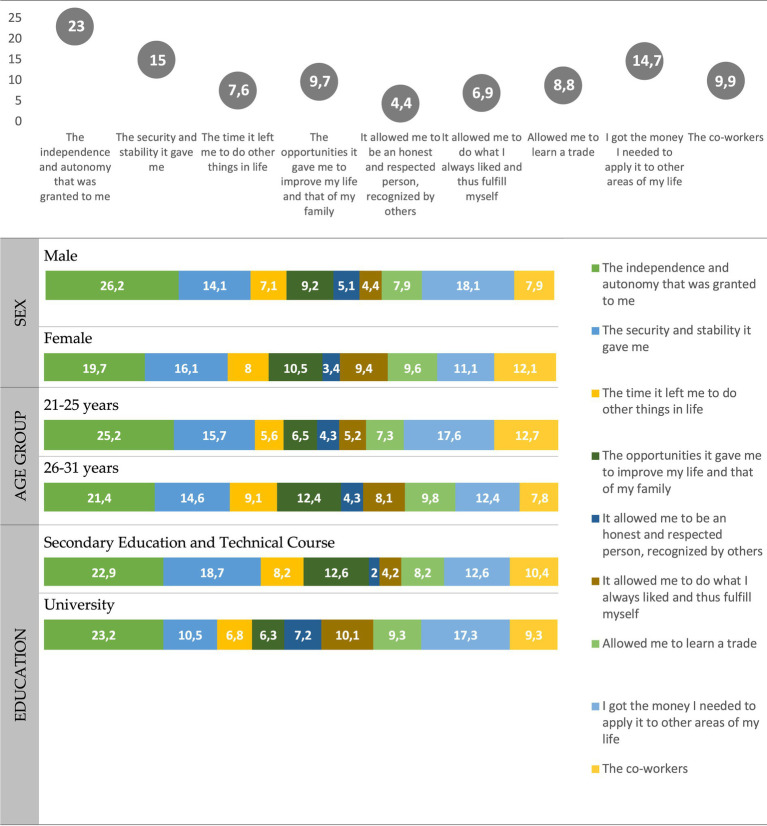
Most important qualities valued in present or past professional life (%). Source: own preparation based on a survey of young people in the Municipality of Braga.

**Figure 6:**
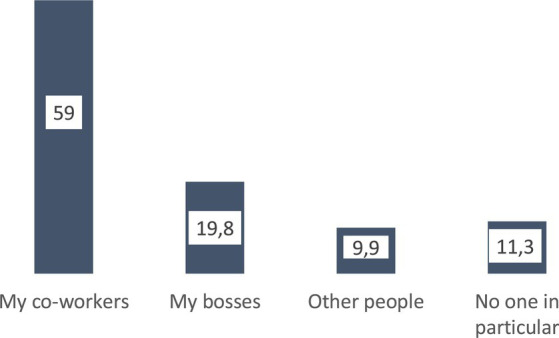
Most important people at work (%). Source: own preparation based on a survey of young people in the Municipality of Braga.

**Figure 7:**
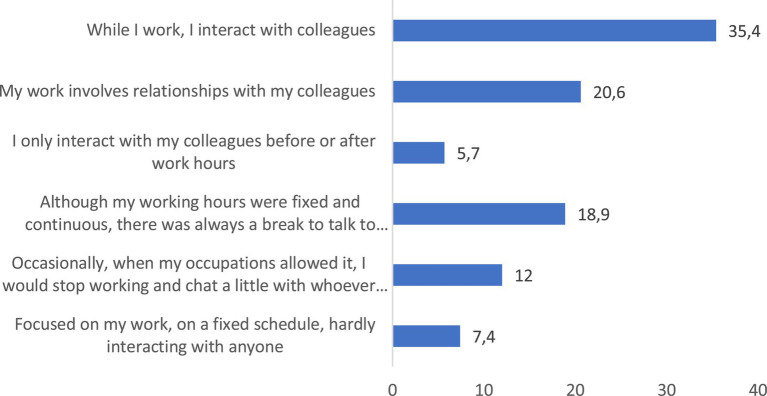
Statement that comes closest to how you spend or spent a day at work (%). Source: own preparation based on a survey of young people in the Municipality of Braga.

What do young people in Braga value most about their work? The qualities that this youth values most, whether they are working or not, is *independence*, important to 23% of those surveyed, followed by *security* and *stability* (15%), and *having enough money to dedicate to other spheres of life* (14.7%). On the contrary, what young people value least about work is that *it allows me to be an honest and respected person, recognized by others*, only important for 4.4% of the people surveyed and that *it has allowed me to do what I always liked and thus be a respected person*, which only 6.9% of those surveyed consider important (Figure 5). Young people who are working also value the *relationship with coworkers* (important for 59% of respondents). For that part of the youth that has some work experience, this assessment is even higher, since 6 out of 10 say that *relationship with co-workers* is important in their daily work day, because either their work involves such interaction (this is what 35.4% of those surveyed say), or they interact with their co-workers while working (as stated by 20.6% of the people surveyed) (Figures 6, 7).

**Figure 8:**
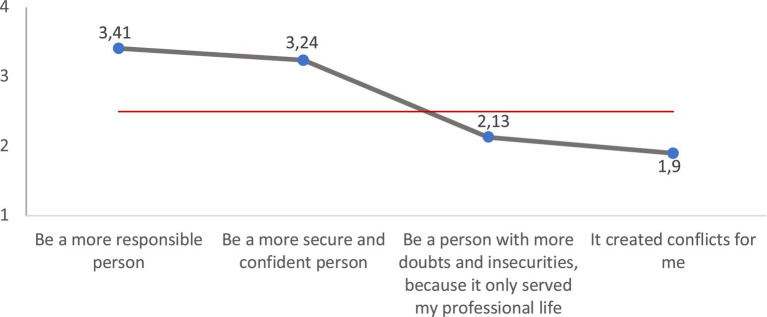
What was transmitted at work is useful for (average). Source: own preparation based on a survey of young people in the Municipality of Braga.

By virtue of this assessment of work, a majority of the young people from Braga that we have surveyed believe that work has helped them become more *responsible, secure and confident* people. (Figure 8). For all these reasons, they say they feel mostly *satisfied with their work* (Table 1). However, those who feel most satisfied, although the difference is not substantial, are those young people who work, or have worked, in a family business, those who have indefinite contracts and those who work for the public administration, whose working conditions, both in terms of salary and stability, are better than those of the rest of the young people employed in the private sector (Table 2). The different academic levels of the youth surveyed barely introduce significant differences with respect to this assessment of work.

**Table 1:** Job satisfaction (average).

Sex		Age group		Education	
Male	3.09	21–25 years old	2.99	Secondary Education and Technical Course	3
Female	3.08	26–31 years old	3.16	University	3.19

Own preparation based on a survey of young people in the Municipality of Braga.

**Table 2:** Job satisfaction (average).

Type of activity	Employee	3.11
	Self-employed	2.83
	Worker in family business or company	3.5
Type of contract	Unlimited duration contract (Permanent)	3.41
	Limited-term contract (Temporary)	2.86
	No contract	2.1
Organization or company for whom one works or worked	Central or local government	3.5
	Other public administration sector (such as education, health, among others)	3.36
	A state company	3.4
	Private sector company	3.2
	Other	2.22

Own preparation based on a survey of young people in the Municipality of Braga.

**Figure 9:**
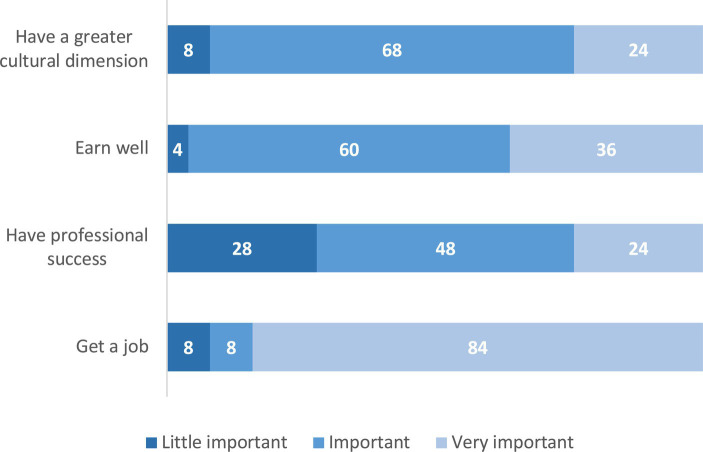
Importance of school training for young people who have never worked (%). Basis: young people who have never worked (*N* = 42). Source: Own preparation based on a survey of young people in the Municipality of Braga.

**Figure 10:**
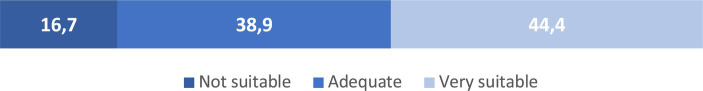
To what extent is the educational training you received suitable for the roles that the job market is currently demanding? (%). Basis: young people who have never worked (*N* = 42). Source: own preparation based on a survey of young people in the Municipality of Braga.

For the majority of young people from Braga that we have surveyed and who have never worked, *school training is very important to find a job* (84%) (Figure 9). A majority of these young people also consider that their academic training is in relation to what the labour market demands (Figure 10), perhaps because they themselves have chosen this training as a path to their labour insertion.

**Figure 11:**
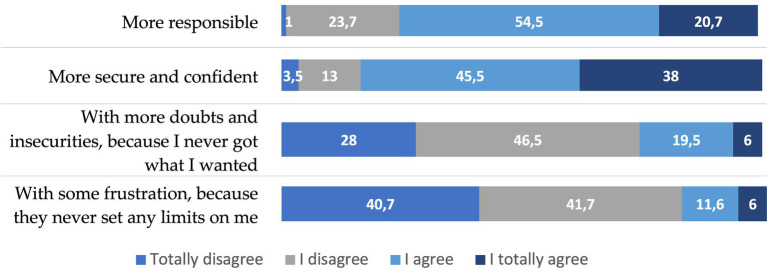
To what extent do you agree that a life of leisure, fun and shopping has made you a person with %. Source: own preparation based on a survey of young people in the Municipality of Braga.

To more adequately contextualize the value that young people from Braga attribute to work, we have introduced into the questionnaire some questions related to consumption, since, as several studies have shown for some decades now, consumption is a fundamental part of the construction of the identity of Portuguese youth (Pais, 2003, 2005; Pais, 2009; Pappámikail, 2011), in a similar way to what happens with other youth in post-industrial societies (Lipovestky, 2007; Bauman, 2010; Cuzzocrea, 2018; Luis et al., 2020). Well, the majority of young people from Braga that we have surveyed, specifically 8 out of 10 (82.4%), say they agree, or totally agree, that their life of leisure, fun and shopping makes them *more responsible, secure and confident* (Figure 11). Now, these purchases are no longer intended to improve the social position of those who make them (only 7% of the people surveyed consider this), but rather to become *more original and interesting people* (34% of those surveyed believe so), and to *improve the quality of life and have a more comfortable life* (47% of respondents chose this answer among those presented to them). As can be seen from the data presented, the young people from Braga that we have surveyed link their leisure and consumption practices to the fact of having a more comfortable and pleasant life, thinking more about the present than about tomorrow. We can consider, therefore, that the sphere of leisure and consumption is closely linked to the construction of the identity of the youth of Braga, compensating and completing that other part of their identity related to the world of work.

In the next section we will discuss how the work identity of the youth of Braga that we have analysed is shaped by the relationship they establish between their work situation and their work culture.

## 4 Discussion

In what follows, we will discuss the results obtained in our research.

As suggested at the beginning of this work, the world of work, and in particular that of the youth of Braga, the object of our research, is shaped as a dense social experience that cannot be analysed taking into account only the task performed and the contractual conditions under which one works, but also taking into account other aspects that constitute said work experience. Starting from the confluence between the work situation and the work culture, the world of work with all its complexities can be understood.

This way of understanding work, as a dense social and cultural experience that is not exhausted by particular material conditions, but rather acquires a certain meaning in relation to the meaning given to it by those who participate in it, is linked to the tradition of classical sociology itself. We find this perspective in Max Weber’s famous essay on the Protestant Ethic and the Spirit of Capitalism, which shows how for Calvinists their work experience did not make sense in relation to the particular task performed, because “God does not require work for the sake of work,” but from the moment in which this task was directed towards a greater purpose, since it was carried out *Ad maiórem Dei glóriam* (Weber, 1998, p. 172–173). Durkheim’s work, especially that of his youth, was also aimed at showing that in capitalist industrial society, work activity could only achieve greater meaning if it was carried out in relation to the values that modernity had proclaimed, those of freedom, equality and social justice (Durkheim, 1995, p. 442–443; Lukes, 1984, p. 338). “We do not realize enough – he wrote - how laborious it is to build this society in which each individual will have the place he deserves and will be rewarded as he deserves, and in which everyone, consequently, spontaneously contribute to the good of each and every one” (Durkheim, 1995, p. 479). Only in this way, Durkheim maintained, would individuals feel called to perform different work functions for a higher interest than the purely material and personal, thus finding greater satisfaction and meaning in their professional activity. Now, this plan was very ambitious, because it sought to make the increase in productivity, a goal towards which industrial society was mainly oriented, compatible with the achievement of a series of individual, social and moral principles.

However, as is known, in the process of development and transformation of capitalist industrial societies these two dimensions - the most economic one and the one linked to a more social and cultural purpose - have frequently been at odds, because the reasons that the industrial system had for people to work did not coincide with those that people had to work, since these reasons were inscribed in the pre-industrial order in the social, cultural and moral spheres. This order is what industrial society had to break with to establish its own work morality (Thompson, 1979, 1995; Pollard, 1987, 235 y ss.; Offe, 1990, p. 80–81; Castel, 2001, 327 y ss.).

In the period after the Second World War, both dimensions were harmonized – the economic and the social, cultural and moral-, since economic growth was accompanied by the full integration of the adult population, initially male, in the world of work through the recognition of a wide range of social rights. As the British sociologist TH Marshall said, in this way each worker had for the first time the opportunity to become a true gentleman, proud of the social position he had thus achieved (Marshall, 1998, p. 18). The Marxist analyses that insisted on seeing this worker as an alienated being, dedicated to the monotonous and fragmented work of the Fordist company, failed to see, carried away by their theoretical and analytical categories, that those who should have been alienated in this way in reality were not, because work had become for them a dense experience, not only material, but also social and moral (Sennett, 2001).

In post-industrial societies, both the forms of work organization and work ethics (Boltanski and Chiapello, 2002) and culture have been transformed (Brooks, 2001; Florida, 2004; Lloyd, 2012; Valenzuela et al., 2015; Farrugia, 2018). In this context, the relationship between work cultures and working conditions has become more complex. All this means that certain more or less precarious working conditions do not necessarily lead to a conception of work that is also precarious, because the old Fordist work morality no longer integrates the current work culture, and particularly that of the youth of post-industrial societies (Durán Vázquez and Duque, 2018; Farrugia, 2018; Balderson et al., 2020). You cannot pour old wine into new vessels. It is from this perspective that we have analysed the world of work of the youth of Braga, trying to understand the relationship that exists between their work situation and their work culture.

The employment situation of this youth can be said to be mostly precarious, because although an important part of the older young people, those between 26 and 31 years old, have indefinite contracts, their salaries are quite low (less than 900 euros per month), and almost a quarter of the youngest, between 21 and 25 years old, do not have any type of employment contract. And, although the level of qualification of this youth has not stopped increasing in recent decades, all this has not translated, as has been shown in this work, into a proportional improvement in their working conditions, which has led to overqualification.

In short, the working conditions of the youth of Braga, and especially those between 21 and 25 years old, are characterized by precarious work contracts with low salaries, which do not require the qualifications that many of these young people possess. All of this means that in many cases they need to supplement their salaries with the resources provided by their families, with whom a good part of them still live. Work is not, therefore, for said youth a source of autonomy and personal freedom. It could be predicted, therefore, that their work attitudes would be rather negative, corresponding to a precarious work situation. Is this so?

Let us observe how these young people value their work. As has been shown, what they value most about their work, whether they work or not, is *independence*, followed by *security* and *stability*, and *having enough money to dedicate to other spheres of life* (Figure 5). These data can surprise us, if we do not interpret them properly. Indeed, although their jobs do not allow independence, if this is understood as living without family help, they are valued precisely for this quality, because independence is not identified here with the old biographical cycle of having one’s own home and be able to start a family, but with having enough *money to dedicate to other spheres of life*. In this sense, *job stability* is also valued, not so much because it allows one to gradually trace a certain biographical trajectory, which precarious jobs hardly make possible, but because it guarantees the opportunity to free oneself in other spheres of life. From this point of view, work acquires an instrumental value. Faced with this assessment of work, it is hardly considered that work *allows one to be an honest and respected person, recognized by others* (Figure 5); because this activity is no longer associated, as in other times, with moral values that express a certain sense of honour and the dignity. Nor to the values of achievement and status (Sennett, 2001, 13 y ss.; Durán Vázquez and Duque, 2018).

What is valued about work are the social relationships it makes possible (Figure 6), an assessment that is in line with what other studies on Portuguese youth have been supporting for at least a decade and a half (Pais, 2005; Pappámikail, 2005, 2011; Durán Vázquez and Duque, 2018). A sociability, which, according to the majority of young people living in Braga surveyed who work, is made possible by their own jobs (Figure 7).

What little stands out among the qualities of the work of the young people from Braga we surveyed is that *it allows them to do what they love most and thus achieve fulfilment* (Figure 5). Indeed, although work is increasingly appreciated, and particularly among young people in post-industrial societies, as a means to develop personal tastes, and less and less in relation to dimensions linked to achievement and certain social and moral duties, such as various studies have shown (Cogin, 2012; Lloyd, 2012; Valenzuela et al., 2015; Samuel and Kanji, 2019; Balderson et al., 2020), for a large part of the youth, and specifically for those from Braga that we have surveyed, who mostly have precarious jobs, or who have the prospect of having them, work cannot be perceived as the expression of personal tastes and vocations. The meaning of work refers, as has been said, increasingly to instrumental aspects, such as salary and stability, which make it possible to *have enough money to dedicate to other spheres of life* (Figure 5), and particularly to those of leisure and consumption, which appears as an important facet of the identity of these young people (Figure 11), just as happens with other youth in European post-industrial societies (Cogin, 2012; Durán Vázquez and Duque, 2018; Hiemer and Andresen, 2019). If the job satisfies any of these aspirations, young people may feel more or less satisfied with the job they do (Tables 1, 2). All this shows that work is no longer such a fundamental part of personal identity, as it had been for youth in other times (Durán Vázquez and Duque, 2018). Precisely for this reason, some young people prefer to work fewer hours, freeing up the remaining time for other activities that have equal or more meaning for them (Eurofound, 2017; Hiemer and Andresen, 2019).

In conclusion, as we stated at the beginning of this article, and as we have shown throughout it, the youth of Braga surveyed do not understand work as an activity that is reduced to the mere task performed, nor to its contractual conditions, but in relation to a series of aspects linked to their life worlds. Therefore, although their objective working conditions may be more or less precarious, this does not translate into the devaluation of their work and personal life. Indeed, precisely because work has ceased to be a central aspect of the identity of this youth, in relation to certain moral dimensions, associated with a certain sense of duty, and social dimensions, related to achievement and status; precisely for this reason, although the jobs of these young people no longer allow them to achieve these objectives, their lives do not lose meaning. Because this sense appears associated with the personal relationships that they can make in the workplace, and with the economic possibilities that work provides, in terms of salary, to build themselves autonomously in those social areas in which this youth aspires to achieve the recognition that work no longer provides them.

Research carried out on the world of work must take all this into account, perceiving work as a dense social experience, in which economic, social and cultural aspects intervene, related both to the world of work itself and to other worlds of life. Therefore, more or less precarious working conditions do not necessarily lead to a precarious meaning of life. Because, although work may be a more or less important part of life, life may not be just work.

Considering everything said up to this point, we consider it necessary to make some recommendations from the point of view of labour and educational policies. Indeed, many labour policies are implemented under the idea that work is practically the only activity through which youth can achieve a fuller life. But, paradoxically, the working conditions of youth, especially those in southern Europe, such as Portugal and Spain, are increasingly precarious, making their respective life projects difficult. However, as has been observed throughout this study, young people do not place the meaning of their lives solely in work, but in other areas of their existence. Therefore, labour policies should consider this entire reality and make working time compatible with other periods of life, in which young people also aspire to be recognized. To achieve this, it is not only essential to put an end to job insecurity, but it must also be considered that since most of the youth consider that not all of life is work, work must not only enable the development of a decent work-life project, but it must also allow this development to extend to other spheres of existence.

Concerning education, we believe that educational policies have ended up being oriented almost exclusively to the changing scenarios of the current world of work. All of this has meant that under the sign of competency-based learning, all academic knowledge that is not oriented towards the acquisition of the work skills required by the world of work has been degraded. Thus, instruction has been confused with education, in such a way that all education has become mere instruction in a series of skills, which are subject to permanent review, without an educational goal to guide them. The consequence of all this is the discouragement of a large part of the youth, for whom their academic careers make less and less sense. It is necessary, therefore, to make a deep reflection on the meaning of school education in our societies. A reflection that establishes what the main goals of education are, separating them from those other more instrumental ones, under the consideration that although education must be for work, not all education must be a preparation for work (Durán and José, 2019; Durán and José, 2021).

## Data availability statement

The raw data supporting the conclusions of this article will be made available by the authors, without undue reservation.

## Author contributions

ED: Writing – original draft, Writing – review & editing. JD: Writing – original draft, Writing – review & editing.
